# Increasing the Sustainability of Maize Grain Production by Using Arbuscular Mycorrhizal Fungi Does Not Affect the Rumen of Dairy Cattle (*Bos taurus*) and Buffalo (*Bubalus bubalis*)

**DOI:** 10.3389/fvets.2020.556764

**Published:** 2020-10-15

**Authors:** Antonella Chiariotti, Joan E. Edwards, Gerben D. A. Hermes, Gennaro Catillo, David Meo Zilio, Sabrina Di Giovanni, Hauke Smidt, Luca Buttazzoni

**Affiliations:** ^1^Council for Agricultural Research and Economics (CREA), Research Center for Animal Production and Aquaculture, Monterotondo, Italy; ^2^Laboratory of Microbiology, Wageningen University & Research, Wageningen, Netherlands

**Keywords:** rumen microbiome, bacteria, archaea, protozoa, anaerobic fungi, fermentation

## Abstract

New approaches are needed to improve the sustainability of feed production and utilization by ruminants. Promising approaches include increased use of buffaloes for more sustainable milk production, and arbuscular mycorrhizal fungi (AMF) to reduce crop production input needs. However, studies assessing the effect of crops grown in the presence of AMF on rumen microbial utilization are limited. Based on current knowledge, we hypothesized that maize grain grown on AMF-inoculated soil affected ruminal fermentation and microbiota, and that this effect differed between buffalo and cattle. A dietary cross-over study (four weeks per diet) was conducted using rumen-cannulated cattle (*n* = 5) and buffalo (*n* = 6) to assess the effect of maize grain (3.9% (w/v) of diet) grown on soil with or without AMF (15 kg/ha) on ruminal fermentation and microbiota. Production of maize on AMF-treated soil did not affect any of the assessed ruminal fermentation parameters, microbial concentrations, or prokaryotic community composition (using prokaryotic 16S rRNA gene sequence analysis). In contrast, host type had numerous effects. Protozoal counts, lactate, total VFA and isobutyrate, were significantly higher in buffaloes compared to cattle. Conversely, butyrate was significantly lower in buffaloes than in cattle. Host type explained 9.3% of the total variation in prokaryotic community composition, and relative abundance of nine amplicon sequence variants significantly differed between host types. These findings indicate that AMF treatment of maize crops has no detrimental impact on the value of the resulting maize grains as a ruminant feed, and provides additional insight into rumen-based differences between cattle and buffalo.

## Introduction

Increasing world population, urbanization, and the growing concern over the environmental impact of animal farming means a long-term global strategy for more intensive and sustainable ruminant production is needed (1). Ruminant livestock are important not only for the production of high quantity and quality animal protein (milk and meat) in human diets, but also their ability to produce this using fibrous feeds that cannot be used by humans (2). Furthermore, low-quality feedstuffs, which contain high levels of fiber and low levels of fermentable carbohydrates, are more efficiently utilized by buffaloes than cows. As such, there is increasing interest in this more sustainable ruminant species for livestock production. This is evidenced by the 15.7% increase in buffalo milk production worldwide compared to 4.09% in dairy cows during 2014–2018 (FAOstat data). In Italy, the increase in the same period was up to 21%. This is because in Italy, buffalo milk is used to produce mozzarella cheese which is the third largest Italian DOP (i.e., protected designation of origin) cheese in terms of market value.

Buffalo are more efficient at utilizing low-quality feedstuffs, compared to cattle, due to differences in their digestive system anatomy and physiology (3). Compared to cattle, buffalo have higher rumen retention time (4) and higher feed digestibility (5–7). Associated with this, rumen microbial concentrations and community composition have also been shown to differ between cattle and buffalo (7–9). In order to decrease the impact of both buffalo and cattle production on the environment, new approaches are also needed to improve the sustainability of feed production, for example by decreasing the need for crop fertilization, pest and weed control, and water input. Mycorrhizal fungi are one means of doing this, as they can decrease crop production costs and associated environmental pollution risk.

Mycorrhizal associations of fungi with plants are present in almost all ecosystems, from deserts to arable land (10). Arbuscular mycorrhizal fungi (AMF) are the most common type of fungi that form a symbiotic relationship with terrestrial plants (11). The mycorrhiza formed essentially result in an extension of the plant's root system (12). AMF obtain carbohydrates from their plant host, and in return they provide the plant with mineral nutrients including phosphorus, nitrogen, and potassium (11, 13). AMF positively influence the accumulation of iron and zinc in the plant, which are among the numerous minerals that are relevant to ruminant livestock nutrition (14–16).

As well as exchanging minerals for nutrients, AMF relieve plants from abiotic stress such as drought, salinity and heavy metals (17–19). Protection of host plants from biotic stresses, such as microbial pathogens, has also been reported (20, 21). Furthermore, AMF can contribute to mycotoxin control (22) and induction of the expression of defense-related genes (23). In broader terms, AMF also improve soil structure (24) and can even play an important role in maintaining plant biodiversity and ecosystem stability (25).

Some commercially available AMF preparations include other biological components, such as plant growth promoting rhizobacteria (PGPR) (26–28). PGPR are very effective in promoting plant growth by releasing growth stimulating hormones, and can also give some protection against soil-borne pathogens (29, 30). With maize, PGPR have been shown to improve the mineral status of the plant, promote mycorrhiza colonization of maize roots and increase maize growth (30, 31). As maize is a basic feed for ruminants, differences in its quality can have a significant impact on milk yield and composition. It has been reported that silages from AMF treated forage crops have a higher protein and dry matter content compared to non-AMF treated forage crops (26, 28). Inoculation with both the PGPR strain *Pseudomonas fluorescens* Pf4 and AMF has also been shown to promote maize growth under field conditions, and can differentially affect grain nutritional content (16). However, only limited studies to date have looked at the effect of AMF applied during the production of feed on livestock.

A study with dairy cows and buffalo fed silages of sorghum and maize produced in the presence of AMF looked at milk production parameters and *in vitro* feed digestibility (28). Another study assessed the effect of AMF on the *in vivo* digestibility of fresh barley and berseem clover in goats (27). Both studies found a positive effect of AMF on forage digestibility, however, neither of these studies directly assessed ruminal fermentation parameters and the associated microbiota. In a preliminary communication, we have reported on a feeding trial with lactating Holstein cows where we evaluated the effect of maize grain grown on mycorrhized soil (32). Differences were found in term of maize grain degradation characteristics and, compared to the control maize grain fed animals, the grain grown on soil inoculated with AMF increased rumen microbial concentrations, dry matter intake and milk protein content.

To date, no other information is available regarding the effect of maize grains grown on soil inoculated with AMF on the rumen microbiota and associated fermentation in cattle, and nothing is known in buffalo. Based on the available knowledge outlined above, we hypothesized that maize grain grown on soil inoculated with AMF affects ruminal fermentation and associated microbiota, and that this effect will differ between buffalo and cattle. In order to test this hypothesis, a dietary cross-over study was conducted using rumen-cannulated Holstein Friesian dairy cows and Mediterranean buffaloes. Ruminal fermentation parameters and rumen microbial concentrations were measured, and barcoded amplicon sequencing of the 16S rRNA gene was used to assess rumen prokaryotic community composition.

## Materials and Methods

### Animals and Experimental Design

Rumen cannulated, non-lactating Mediterranean buffalo (*Bubalus bubalis*) cows (*n* = 6) and Holstein-Friesian (*Bos taurus*) dairy cows (*n* = 6) were used for the trial. Animals were kept in paddocks, with three animals of the same host type in each paddock. Animals were fed the same basal diet for 4 weeks prior to the start of the trial, which had a crossover design comprised of two experimental periods. Within the first experimental period, half of the animals for each host type were assigned to the diet containing maize grain produced with AMF, and the other half were assigned to the diet containing maize grain produced without AMF. These experimental diets were then fed for 4 weeks. After this, the basal diet was fed again for 4 weeks as a “washout” period before start of the second experimental period. In the second experimental period, diets were then switched over within the same host type, and were fed for 4 weeks.

Both host type groups started the study with six animals, however, due to one Holstein-Friesian cow having to be euthanized during the first experimental period the number of cattle was subsequently decreased to *n* = 5. Rumen sampling was performed during the last 3 consecutive days of each experimental period. Statistical analysis was performed using animal replication, i.e., for cattle *n* = 5, and for buffalo *n* = 6.

The animal study research protocol was approved by the National Ethics Committee (Ministry of Health Decree 26/2014, authorization n°399/2018, Italy) in accordance with the guidelines established by the EU Council/Directives 86/609/EEC.

### Maize Grains and Diet

Mycorrhized soil maize grains (M) as well as control maize grains (C), were both produced on two farms in a maize cropping area in the northern Italy near Cremona. For only the mycorrhized soil produced maize grains, Micosat ® Cereal, was applied (15 kg/ha) as a granular preparation when the maize crop was sowed according to the manufacturer's recommendations. The Micosat® Cereal product is a biostimulant microbial consortium consisting mainly of 40% AMF species (*Glomus caledonium* GM 24, *G. coronatum* GU 5, and *Rhizophagus irregularis* RI 31) in the form of ground spores, hyphae, and root fragments of donor plants, along with 9.3% (2.0 × 10^7^ C.F.U./g) of both PGPR (*Bacillus subtilis* BR 62*, Streptomyces* sp. ST 60*, Paenibacillus durus* PD 76) and saprophytic fungi (*Trichoderma harzianum* TH 01*, Trichoderma atroviride* TA 28). The composition of the remainder of the product is abiotic (C.C.S. Aosta s.r.l., Quart, AO, Italy). The rhizobacteria and the fungi are added by the producer, as they are thought to have additional value alongside AFM.

Diets containing the maize grains (Diet M or Diet C) were fed once per day (11 am) as a total mixed ration (TMR) with the same proportion of all ingredients for both species, and each group receiving 60 kg feed/day (i.e. 20kg/head/day on average). Feed refusals were not collected (it was observed during the study that feed refusals were all generally <1kg for both host types), and consequently dry matter intake (DMI) was not assessed or predicted in this study. After the loss of the Holstein-Friesian cow from the trial, the corresponding group then received 40 kg feed/day. Details of the composition of the TMR and basal diet are provided in the Supplementary Methods. Dry matter and chemical analysis of the basal diet, maize grains and the associated TMR diets (Table 1) were performed according to the methods described in the Supplementary Methods. Buffalo and cattle nutritional requirements are provided in Supplementary Tables 1 and 2, respectively.

**Table 1:** Dry matter (g/kg) and chemical analysis (g/kg DM) of the basal diet, maize grains and the associated total mixed ration (TMR) diets.

**Item**	**Dry matter**	**Crude protein**	**Crude fiber**	**Ether extract**	**Starch**	**Ash**	**NDF^**#**^**	**ADF^**#**^**	**ADL^**#**^**	**Hemi-cellulose**	**Cellulose**
Basal diet	609.1	95.8	187.1	22.4	170.8^*^	55.9	427.6	230.7	34.6	197.0	196.0
Maize grain M	881.7	89.1	26.9	36.6	696.4	12.6	158.1	39.7	16.1	118.4	23.6
Maize grain C	882.8	92.3	30.8	39.8	711.1	12.6	145.6	44.5	15.7	101.1	28.8
TMR M	556.9	157.9	213.7	33.0	298.4^*^	62.8	447.8	259.7	44.3	188.1	215.4
TMR C	557.6	158.9	214.3	33.5	299.0^*^	62.8	445.7	260.4	44.2	185.2	216.2

*Maize grain M, grown on AMF treated soil*.

*Maize grain C, grown on untreated soil*.

*TMR C, diet containing maize grain grown on untreated soil*.

*TMR M, diet containing maize grain grown on AMF treated soil*.

# *NDF, neutral detergent fiber; ADF, acid detergent fiber; ADL, acid detergent lignin*.

* *Non-Structural Carbohydrates*.

### Rumen Sampling

Rumen digesta samples (1 L) were collected by hand from each animal 1 h before morning feeding on 3 consecutive days. Sampling was done from the dorsal and ventral sac of the rumen via the rumen cannula, and the samples pooled. Two aliquots from each rumen sample were made. The first aliquot was strained through three layers of cheesecloth and used for direct determination of pH and protozoa counts. The second aliquot was bag filtered (BagPage. Interscience, France) and treated with a homogenizer for 5 min (Stomacher, VWR International, Pennsylvania, USA). Aliquots of the homogenate were then processed as follows for later analysis: frozen at −80°C for microbial analysis, preserved with sulfuric acid and stored at −20°C for ammonia, volatile fatty acids (VFA) and lactate analysis.

### Fermentation Analysis

Fermentation analysis was performed on all the rumen samples, i.e., 2 experimental periods × 11 animals (5 cows + 6 buffalo) × 3 days. Metabolic fermentation products (i.e. VFA and lactate) were analyzed using high-performance liquid chromatography (HPLC). The procedure is described in detail in the Supplementary Methods. Ammonia samples were analyzed according to the 4030-method reported in the “Analytical Methods for Water” (33).

### DNA Extraction

DNA was extracted from the rumen homogenate samples using a protocol involving a combination of bead beating, Stool Transport and Recovery (STAR) buffer (Roche Diagnostics Nederland BV, Almere, The Netherlands) and the Maxwell ® 16 Instrument (Promega, Leiden, The Netherlands). The procedure is described in detail in the Supplementary Methods.

### Microbial Concentrations

Analysis of microbial concentrations was also performed on all the rumen samples as reported for fermentation analysis. Protozoa were counted using a Fuchs-Rosenthal chamber according to the Warner procedure (34). For absolute quantification of bacteria and archaea, SYBR green based qPCR assays were performed on DNA extracts using a CFX384 Touch™ Real-Time PCR Detection System (Bio-Rad Laboratories BV, Veenendaal, Netherlands) as previously described (35). All qPCR analyses were carried out in triplicate with a reaction volume of 10 μL, which contained 2 μL of 1:50 dilutions of sample DNA extracts. Standard curves (10^2^-10^8^ copies/μL) were generated using serial dilutions of custom synthesized DNA prepared from known 16S rRNA gene sequences of *Ruminococcus albus* and *Methanobrevibacter olleyae* (accessible under ENA accession numbers: CP002403.1 and CP014265.1, respectively).

For absolute quantification of anaerobic fungi, a 5.8S rRNA gene Taqman probe based method was used as previously described (36) with the exception that a CFX384 Touch™ Real-Time PCR Detection System (Bio-Rad Laboratories BV) was used. All qPCR analyses were carried out in triplicate in a reaction volume of 10 μL, which contained 2 μL of 1:50 dilutions of sample DNA extracts. The standard curve (10^1^-10^8^ copies/μL) for the assay was generated using serially diluted custom synthesized DNA prepared from a known partial sequence of the *Neocallimastix* sp. CF17 *rrn* operon that contains the full 5.8S rRNA gene (accessible under ENA accession number: GU055516.1).

### Prokaryotic Community Composition Analysis

Samples from the first day of rumen sampling, for each animal and period, were used for prokaryotic community composition profiling by amplification and barcoding (37) of the V4 variable region of the 16S rRNA gene using the barcoded primers 515F (Parada): 5′-GTGYCAGCMGCCGCGGTAA-3′ (38) and 806R (Apprill): 5′ GGACTACNVGGGTWTCTAAT-3′ (39).

For each sample, triplicate PCRs were performed in a volume of 50 μL containing 1 × HF buffer (Finnzymes, Vantaa, Finland), 1 μL dNTP Mix (10 mM; Promega Benelux, Leiden, Netherlands), 1 U of Phusion® Hot Start II High-Fidelity DNA polymerase (Finnzymes), 500 nM of barcoded primers and 2 ng of template DNA. Cycling conditions consisted of an initial denaturation at 98°C for 30 s followed by 25 cycles of 98°C for 10 s, 56°C for 10 s, and 72°C for 10 s, and a final extension at 72°C for 7 min. PCR success and product size was assessed by agarose gel [2% (w/v)] electrophoresis with 1 × SYBR® Safe (Thermo Scientific).

Triplicate reactions were pooled, purified using HighPrep™ (MagBio Europe Ltd, Kent, United Kingdom), quantified using a Qubit dsDNA BR Assay Kit (Thermo Scientific) and subsequently mixed in equimolar amounts. In order to assess the resulting data quality and potential effects of technical biases, a non-template control (NTC) reaction and a defined synthetic mock community of known composition (37) were included in the equimolar pool. The resulting library was sent for adaptor ligation and 150 nt paired-end sequencing on an Illumina HiSeq4000 instrument (GATC-Biotech, Konstanz, Germany, now part of Eurofins Genomics Germany GmbH).

The resulting raw data was processed with NG-Tax 2.0 using default settings (40). The processing was performed as follows. Paired-end libraries were demultiplexed using read pairs with valid and perfectly matching barcodes. Amplicon sequence variants (ASVs) were then picked. ASVs are defined as individual sequence variants, rather than a cluster of sequence variants with a shared similarity above a user specified threshold as was traditionally the case for operational taxonomic units. ASV selection used an open reference approach as follows. Sequences were ordered by relative abundance for each sample independently, and sequences were considered valid when their relative abundance was >0.1%. Initially discarded low-abundance sequences were then added to ASVs in the dataset, allowing for a single nucleotide mismatch. Taxonomy was assigned using the SILVA 16S rRNA gene reference database version 132 (41). The raw sequence data generated in this study is deposited in ENA under study accession number PRJEB37928, with individual sample accession numbers ranging from ERS4525212-ERS4525234.

### Statistical Analysis

The microbial concentration and fermentation data were analyzed using the PROC GLM procedure of SAS 9.4 software (42) using the following univariate linear effects models: Y_ijkml_ = μ + A_i_ + B_j_ + C_k_ + (AB)_ij_ + a_m_ + e_ijkml_ where: Y is the observation vector for the all traits; μ is total average for the trait; A_i_ is the fixed effect of species: buffalo or cattle; B_j_ is the fixed effect of diet: M or C; C_k_ is the fixed experimental period (AB)_ij_ is the interaction between species and diet; a_m_ is the animal modeled as random effect; e_ijkl_ is the random residual. The statistical significance of all traits and least-squares means were determined using Student's *t*-test in the GLM procedure. A probability value <0.05 was considered significant.

Prokaryotic community composition data was analyzed in R version 3.4.0 (43). Stacked bar graphs of individual microbiota composition were created by summarizing the relative abundance of all ASVs to either the phylum level, or to the top 15 most abundant prokaryotic genus level groupings. A stacked bar graph of the average microbiota composition of the treatment groups was also created using the summarized relative abundance of all ASVs to the top 20 most abundant prokaryotic genus level groupings. In terms of both genus level graphs, for clarity all other genus level groupings were summarized as “Other.” Pairwise distance between microbiota samples (beta-diversity) was visualized with Non-metric Multidimensional Scaling (NMDS). The NMDS algorithm places each sample into a two-dimensional space such that the between-sample distances are preserved as well as possible. The weighted UniFrac and unweighted UniFrac metrics, which are based on the phylogenetic relatedness of the ASVs, were used as estimator of microbial beta diversity between the animals. While weighted UniFrac considers the abundance of each ASV, unweighted UniFrac provides equal weight to all ASVs, thereby giving equal importance to presence or absence of low and high abundance ASVs. Redundancy Analysis (RDA) was performed to determine the multivariate effects of the environmental variables on the microbiota composition using the *rda* function from the *vegan* package (44). RDA is a gradient technique summarizing the linear relationships between a set of variables i.e., prokaryotic community composition and a set of explanatory variables such as host type, dietary intervention, and experimental period. ASV abundance was normalized by centered log ratio scaling to account for compositionality of the data (45). To determine the effect sizes and overlap of the significant environmental variables, variation partitioning was performed using the *varpart* function from *vegan*.

## Results

### Ruminal Fermentation and Microbial Concentrations

For all of the analyzed ruminal fermentation parameters there was no significant effect of diet (Table 2). However, some of the ruminal fermentation parameters were affected by host type (Table 2). Lactate concentrations were significantly (*P* = 0.04) higher in buffaloes compared to cattle. Total VFA concentrations showed the same trend, being significantly (*P* < 0.001) higher in buffaloes compared to cattle. The molar proportion of isobutyrate was also significantly (*P* = 0.02) higher in buffaloes compared to cattle. In contrast, the molar proportion of butyrate was significantly (*P* = 0.02) lower in buffalo compared to cattle. No other ruminal fermentation parameters were significantly affected by host type (Table 2).

**Table 2:** Effect of diet and host type on ruminal fermentation parameters and microbial concentrations.

	**Diet**^**§**^		**Host**			***P*****-values**	
	**C**	**M**	**Cattle**	**Buffalo**	MSE^*^	**Diet**	**Host**
							
pH	6.5	6.4	6.5	6.4	0.37	0.30	0.97
Ammonia (mg/dl)	30.6	29.9	28.9	31.6	0.09	0.76	0.25
Lactate (mM)	0.2	0.2	0.1	0.3	0.33	0.65	0.04
Total VFA (mM)	117.3	123.2	88.7	151.8	50.28	0.65	<0.001
Molar proportions:							
Acetate	66.3	65.5	63.0	68.8	12.34	0.82	0.07
Propionate	14.9	17.7	16.0	16.6	6.89	0.12	0.77
Isobutyrate	1.1	1.3	0.5	1.9	2.41	0.77	0.02
Butyrate	10.4	11.7	12.6	9.6	4.92	0.31	0.02
Isovalerate	2.3	2.4	2.3	2.3	1.23	0.89	0.96
Valerate	0.6	1.3	1.3	0.7	1.88	0.13	0.17
Acetate/propionate	4.3	4.4	4.3	4.4	1.60	0.87	0.73
Bacteria^#^	11.0	10.9	10.9	11.0	0.10	0.27	0.40
Anaerobic Fungi^#^	9.1	8.7	8.9	8.9	1.18	0.27	0.87
Archaea^#^	10.4	10.3	10.3	10.4	0.11	0.21	0.12
Protozoa^$^	4.0	4.4	3.4	4.9	2.22	0.52	0.01
Archaea:bacteria	0.9	0.9	0.9	0.9	0.01	0.72	0.34

§ Diet C contained maize grain grown on untreated soil. and Diet M contained maize grain grown on AMF treated soil.

* *Mean Standard Error*.

# *Values are expressed as Log10 gene copies per g rumen sample. For bacteria and archaea this is the 16S rRNA gene. and for anaerobic fungi the 5.8S rRNA gene*.

$ *Protozoa cell counts are expressed as ×10^5^ cells/ml filtered rumen sample*.

As with the ruminal fermentation parameters, diet had no significant effect on any of the analyzed rumen microbial concentrations or the archaea/bacteria ratio (Table 2). Host type had a significant (*P* = 0.01) effect on protozoal counts, being higher in buffaloes compared to cattle (Table 2). No other microbial concentrations or ratios were affected by host type (Table 2).

### Prokaryotic Community Composition

**Figure 1:**
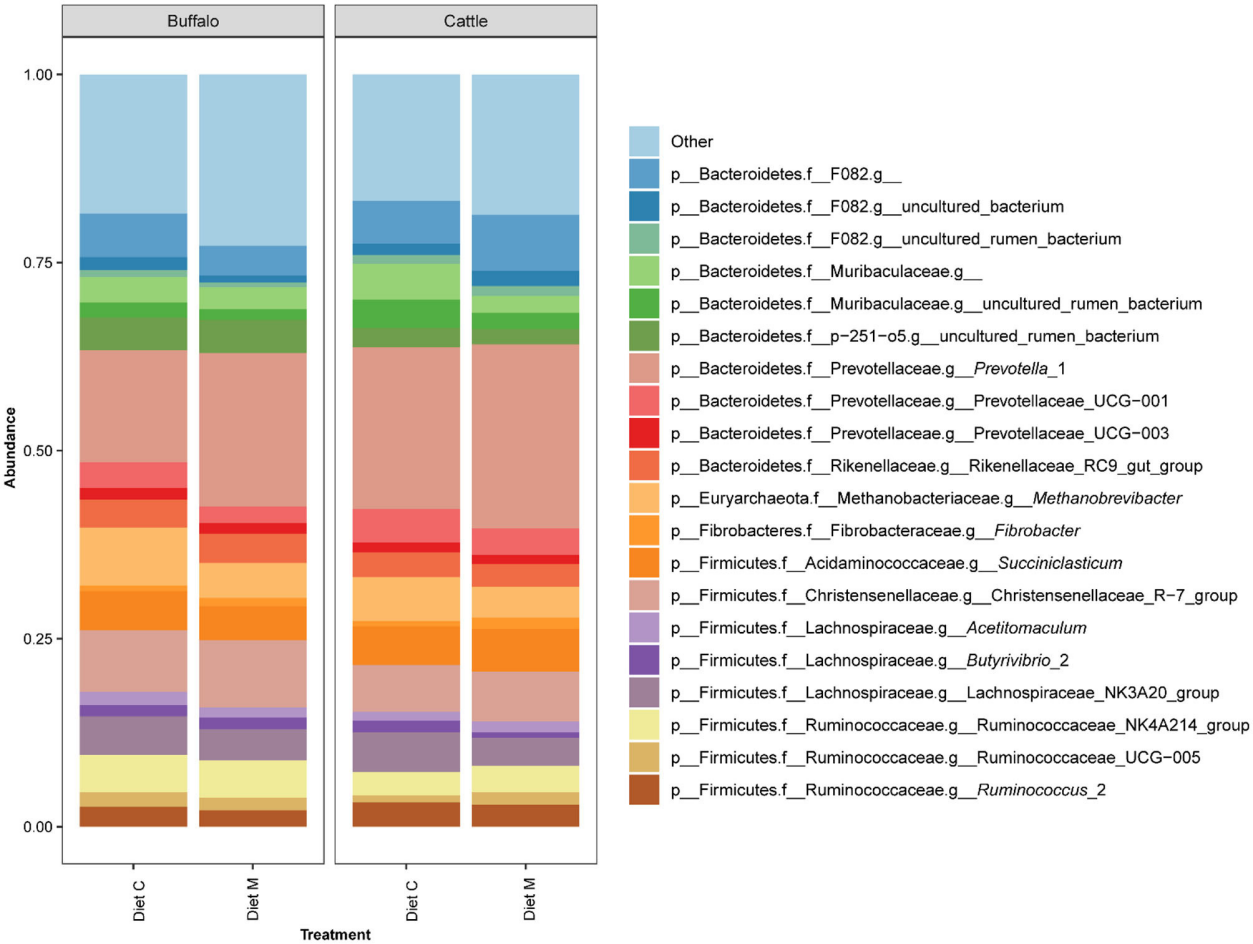
Bar graph showing the average of the 20 most abundant genus level groupings of ASVs from the rumen microbiota of Holstein-Friesian cattle (Cattle) and Mediterranean buffalo (Buffalo) fed diets that differed in terms of containing maize grain which was grown on soil that was treated (Diet M) or not (Diet C) with a commercial AMF preparation.

Of the 960 bacterial and archaeal ASVs detected in the dataset, only three could not be annotated by version 132 of the SILVA database. The number of ASVs annotated at the different taxonomic ranks were as follows: kingdom, 957; phylum 957; class 956; order, 955; family 945; genus 840. The two bacterial phyla Bacteroides and Firmicutes accounted for the majority of the prokaryotic community composition at the phylum level (Supplementary Figure 1). The 20 most predominant (>1.5% mean abundance) genus level groupings of the ASVs in the dataset belonged to the phyla Firmicutes, Bacteroidetes, Fibrobacteres, and Euryarchaeota (Figure 1). Among these, genus level groups within the Firmicutes were annotated as belonging to *Succiniclasticum, Acetitomaculum, Butyrivibrio* 2, Christensenellaceae R−7 group, Lachnospiraceae NK3A20 group, Ruminococcaceae NK4A214 group, Ruminococcaceae UCG−005 and *Ruminococcus* 2. Those within the Bacteroidetes were annotated as belonging to *Prevotella* 1, Prevotellaceae UCG−001, Prevotellaceae UCG−003, Rikenellaceae RC9 gut group and to families F082, Muribaculaceae and p−251–o5. Within the phyla Fibrobacteres and Euryarchaeota, only the genera *Fibrobacter* and *Methanobrevibacter*, respectively, were represented within the top 20 genus level groupings. Within each treatment group, variation between individual animals was observed at both the phylum and genus levels (Supplementary Figures 1, 2).

Weighted and unweighted analysis of beta diversity using unsupervised NMDS showed no separation of samples by diet, and only separation of host type in the unweighted analysis (Supplementary Figure 3). This indicated that differences between host types was largely due to low abundance taxa unique to one host type.

**Figure 2:**
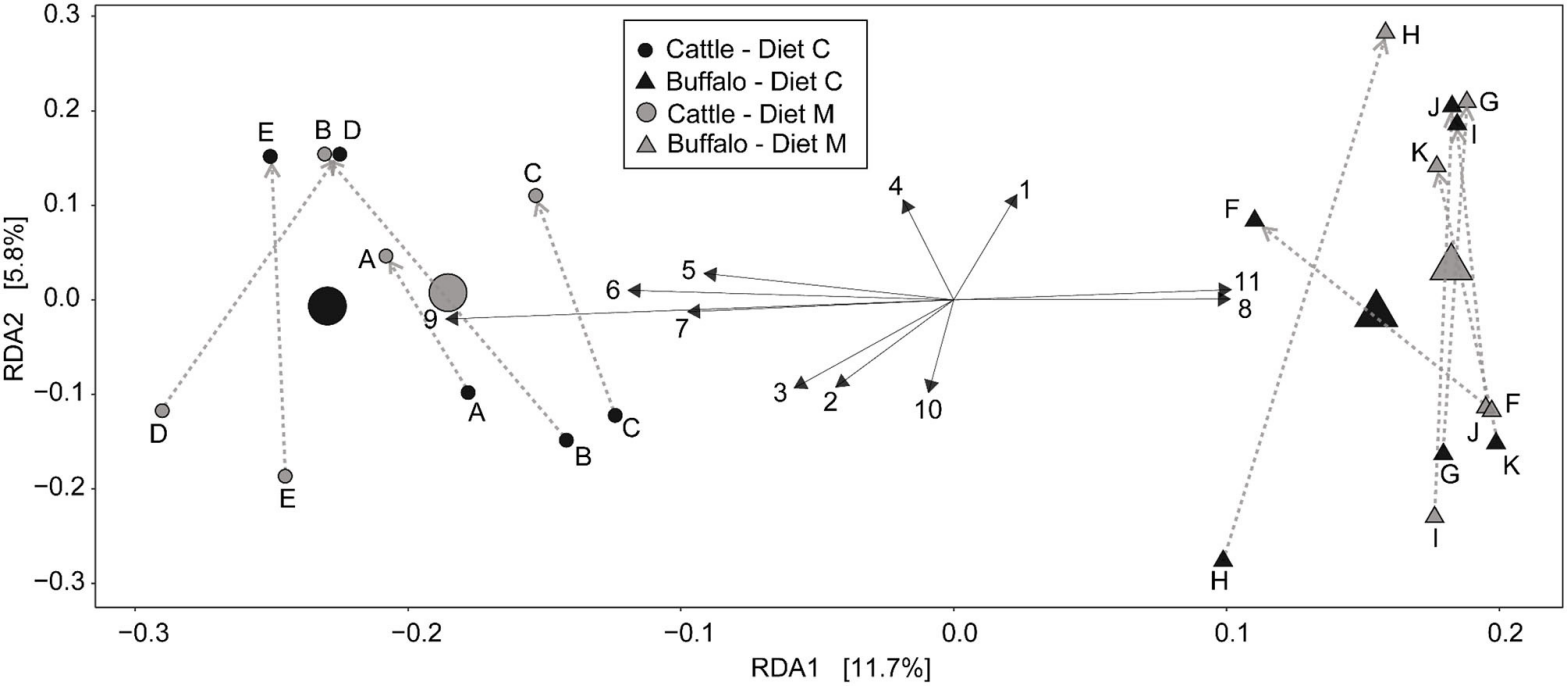
Redundancy analysis biplot of the rumen prokaryotic communities at the level of individual ASVs. constrained by host type (cattle or buffalo) and experimental period. Small symbols are data points represent of rumen microbial communities of individual samples and are labeled with the respective animal codes for the Holstein-Friesian cattle **(A–E)** and Mediterranean buffalo (animals **F–K**). Large symbols indicate the weighted center of all samples from a group. The diets containing maize grains grown on soil that was treated (Diet M) or not (Diet C) with a commercial AMF preparation are also indicated. Gray dotted arrows indicate the direction of the cross-over from the first to the second experimental period. Black solid arrows depict the 11 ASV's whose abundance most accurately fits the shown variation. The longer the arrow. the stronger the association with the samples in that direction. Due to space restrictions on the plot these arrows are numbered and the corresponding ASV identification number and taxonomic annotation is stated in this legend. Annotation is only given to the taxonomic level [i.e., phylum (p), class (c), order (o), family (f), or genus (g)] to which it can be reliably annotated. The ASV details are: [1] ASV 350279275 = f_Prevotellaceae.g_; [2] ASV 35027951 = g_Prevotellaceae_UCG-001; [3] ASV 35027962 = g_Prevotellaceae_NK3B31_group; [4] ASV 350279171 = g_Prevotella_1; [5] ASV 3502796 = g_Prevotella_1; [6] ASV 35027983 =p_Bacteroidetes.f_F082.g_; [7] ASV 3502793 = p_Bacteroidetes.f_F082.g_; [8] ASV 35027920 = p_Bacteroidetes.f_p-251-o5. g_uncultured _bacterium; [10] ASV 35027993 = g_Christensenellaceae_R-7_group; [11] ASV 350279543 = g_Lachnospiraceae_XPB1014_group.

Redundancy analysis (RDA) was performed with ASV level data (i.e., not grouped at the genus level) (Figure 2) to determine the multivariate effects of the experiment on rumen prokaryotic community composition. In contrast to diet (*P* = 0.9), host type significantly affected rumen prokaryotic community composition and explained 9.3% of the total microbiota variation (*P* < 0.001). The difference in host type can be clearly observed in the RDA biplot (Figure 2). Of the 11 plotted ASVs, of which the variation in relative abundance was most accurately fitted with the model, two (numbered 8 and 11 in Figure 2) were positively associated with buffalo. Four of the 11 plotted ASVs were positively associated with cattle (numbered 5, 6, 7, and 9 in Figure 2). Subsequently, an unpaired Wilcoxon test was used to identify ASVs the relative abundance of which was significantly different between host types. Nine of the 960 ASVs (i.e., 0.94%) were significantly affected by host type (Table 3). Four of the nine had a higher mean relative abundance in buffalo compared to cattle, and these belonged to the genera *Butyrivibrio, Prevotella* 1, *Methanobrevibacter* and the Bacteroidetes unclassified family F082. The other five had a lower mean relative abundance in buffalo compared to cattle, and these all belonged to the Bacteroidetes phylum: two ASVs belonged to *Prevotella* 1 and the others belonged to the families Muribaculaceae, p-251-o5 and F082.

**Table 3:** The effect of host type on ASVs was compared using an unpaired Wilcoxon test with data normalized using relative abundance and P values adjusted using the Benjamini–Hochberg procedure to decrease the false discovery rate.

**ASV identification number**	**Assigned Taxonomy**	% mean change in relative abundance in Buffalo compared to Cattle^*^	**Adj. *P*-value**
350279105	p__Firmicutes.f__Lachnospiraceae.g__*Butyrivibrio*_2	0.31	0.006
35027920	p__Bacteroidetes.f__p-251-o5.g__uncultured_bacterium	−0.97	0.009
35027983	p__Bacteroidetes.f__F082.g__	−0.35	0.015
350279131	p__Bacteroidetes.f__Prevotellaceae.g__*Prevotella*_1	0.20	0.034
35027944	p__Bacteroidetes.f__Prevotellaceae.g__*Prevotella*_1	−0.34	0.036
35027973	p__Bacteroidetes.f__Prevotellaceae.g__*Prevotella*_1	−0.54	0.036
35027977	p__Bacteroidetes.f__Muribaculaceae.g__uncultured_rumen_bacterium	−0.40	0.036
350279541	p__Euryarchaeota.f__Methanobacteriaceae.g__*Methanobrevibacter*	0.23	0.036
350279322	p__Bacteroidetes.f__F082.g__	0.20	0.036

* *A positive value indicates that the relative abundance of the ASV was higher in buffalo and a negative ASV indicates that it was higher in cattle*.

As well as host type, experimental period also had a significant (*P* = 0.012) but small affect on the prokaryotic community compostion, explaining 3.1% of the total microbiota variation. The effect of the experimental period is evident from the uniform direction of the arrows which depict time (Figure 2). Independent of the dietary intervention, samples from the first and second experimental period are located toward the bottom and top half of the plot, respectively.

## Discussion

Based on current knowledge, it was hypothesized that maize grain grown on soil inoculated with AMF would affect ruminal fermentation and associated microbiota, and that this effect would differ between buffalo and cattle. This study clearly showed that presence of AMF during production of maize grains had no effect on either host type in terms of ruminal fermentation parameters, microbial concentrations or prokaryotic community composition. This is in contrast to findings of our previous study, with lactating dairy cows, where rumen bacterial and protozoal concentrations were found to increase (32). However, the findings of the current study is perhaps not surprising considering the limited differences that were observed in the chemical composition of diets M and C (Table 1). The lack of effect of the commercial AMF soil treatment on the resulting ruminal utilization of the maize grains indicates there are no issues associated with the use of these grains in ruminant feed.

The maize grain, however, is only one part of the maize crop. Maize silage for ruminants is normally prepared using the whole plant. However, AMF inoculation has been reported not to affect subsequent maize silage composition, and when fed to dairy cows also had no impact on milk yield (28). Similarly, no impact of grain produced on soil inoculated with AMF on milk yield was found in our previous study, although a beneficial increase in milk protein yield did occur (32). This would suggest that irrespective of which part of the plant is used as a ruminant feedstuff, AMF inoculation of maize crops has no subsequent deterimental impact on ruminal processes or ruminant productivity. However, as in this study the commercial AMF preparation used also contained some other biostimulating microbes, comparisons between studies that use commercial products containing AMF and those that use only AMF should be made cautiously.

Host type affected protozoal concentration as well as prokaryotic community composition. Differences between cattle and buffalo have been previously reported in terms of ruminal fermentation, microbial counts, rumen degradability, and digestibility of CP and hemicellulose (3, 5, 7, 8, 46). Longer retention time of feed particles in the rumen has also been reported for buffalo bulls compared to Friesian bulls (4). As ruminal feed retention time of feed influences its ruminal digestion (due to having more time to be degraded by the rumen microbiota), buffaloes are likely to degrade nutrients more extensively than cattle (5). Whilst DMI was not measured in this study, it is likely that differences in DMI reported previously (32) contributed to the higher VFA concentration of buffaloes. However, differences in feed intake pattern between the host types over the day may also be a contributing factor.

It is also important to consider that in this trial the cattle's diet was used for the buffalo, which is richer in nutrients relative to the buffalo's dietary requirements (see Supplementary Table 1). The significantly higher ruminal total VFA concentration in buffalo, compared to cattle, is similar to other data reported by Parmar et al. (47) where buffalo were also given a high concentrate diet. However, despite the higher VFA concentration in buffalo, we did not observe an associated decrease in pH. Buffalo fed the same basal diet on the research farm where this study was conducted usually have a ruminal pH between 6.7 and 7. This pH is higher than was observed in this study. As such, there may well have been a reduction in the buffalo rumen pH during the study due to the high concentration of VFA.

Buffaloes were found in this study to have a higher lactate concentration and molar proportion of isobutyrate. The 3-fold higher concentration of lactate in buffalo compared to cattle in this study is likely to be due to the more efficient ruminal feed degradation in buffalo. Wanapat and Pimpa (48) reported that lactate in the rumen liquid increased significantly with the increase in the dietary proportion of concentrate. According to Nikolov (49), high lactate concentration in the rumen can occur when it is not completely metabolized to propionate. The animal attempts to neutralize it by increasing buffering capability by enhancing salivation. This is normally also associated with increasing amounts of ammonia in the rumen, as was also observed in this study. The increased lactate concentration was not associated with a decreased pH relative to the cattle fed the same diet, and there was no evidence of the buffalo suffering from subacute ruminal acidosis (49).

Branched-chain VFAs such as isobutyrate are required by many rumen bacteria for protein synthesis, particularly cellulolytic bacteria (50). Isobutyrate supplementation of cattle diets has been reported to improve nutrient utilization and ruminal fermentation characteristics (51). As such, it is speculated that the higher molar proportion of isobutyrate in buffalo, compared to cattle, positively affects their ruminal degradation of feed.

The finding of a significantly lower butyrate molar proportion in buffalo, compared to cattle, was consistent with a previous study that compared swamp buffalo with beef cattle (7). Furthermore, it has also been reported that water buffalo have a lower ruminal butyrate concentration relative to Jersey cows (9). The reason for the increased butyrate molar proportion in cattle in this study may be due to it being produced by one or more of the bacteria represented by the five ASVs that were significantly increased in cattle compared to buffalo. However, in this study this cannot be confirmed as the Bacteroidetes F082 and p-251-o5 families are uncultured, fermentation products of members of the Muribaculaceae family have not yet been described (52) and ruminal *Prevotella* spp. do not produce butyrate (53).

It is interesting to note that one of the ASVs annotated as the butyrate producing genus *Butyrivibrio* 2 was found to be significantly decreased in cattle compared to buffalo. This is the opposite from what would be expected based on the butyrate molar proportion data. However, due to the higher total VFA in buffalo, there is actually more butyrate production in buffalo compared to cattle.

Three other ASVs belonging to the genera *Prevotella* 1, *Methanobrevibacter*, and the Bacteroidetes unclassified family F082 were also significantly increased in buffalo compared to cattle. ASVs from *Prevotella* 1 and the Bacteroidetes F082 family were also significantly increased in cattle. This indicates that within this genus and uncultured family significant ecological diversity exists with respect to bovine host type. *Prevotella* spp. are known to be involved in protein degradation and polysaccharide digestion (54), however, the ruminal role of the Bacteroidetes unclassified family F082 is not yet known. In contrast, the role of hydrogenotrophic *Methanobrevibacter* in the rumen is well known due to it's central role in ruminal methane production. An increase in *Methanobrevibacter* in buffalo compared to cattle is consistent with the higher VFA production in these animals, which will also result in more hydrogen being available for methane production. Predictive equations have been used to estimate the enteric methane production in buffalo, however, this needs to be directly assessed *in vivo* (55, 56).

In this study, a higher protozoal concentration was found in buffalo, compared to cattle. This result is consistent with findings of other studies (9, 57). As methanogenic archaea are extra- and intra-cellulary associated with protozoa, this may also explain the higher relative abundance of *Methanobrevibacter* in buffalo compared to cattle. Although the protozoal community composition was not analyzed in the present study, it has been previously shown to differ between water buffalo and Jersey cows (9). In sheep, increased protozoal concentrations have been associated with a decreased rumen bacterial concentration (due to their bacteriolytic activity) and increased ammonia and VFA concentrations (58). However, in our study no differences between buffalo and cattle were found in terms of ammonia or bacterial concentrations. It is now realized though that the role of protozoa in the rumen is more complex than previously thought, as indicated by a review which included a meta-analysis of over 20 different defaunation studies (59).

As well as host type effects on the rumen prokaryotic composition, an effect of experimental period was evident. The reason for this is not clear, and may be related to environmental factors. Temperature could be an indirect trigger to shift rumen community composition, via changes in the physiology of animals in response to heat stress (60, 61). For example, the animal housing area was not temperature controlled and the ambient temperature in the first experimental period (23.9 + 1.7°C) was lower than that of the second experimental period (27.6 + 1.0°C). Regardless though, this finding confirms the importance of using a cross-over design when comparing different diets within the same animals.

## Conclusion

Based on current knowledge, it was hypothesized that maize grain produced in the presence of AMF would affect ruminal fermentation and associated microbiota, and that this effect would differ between buffalo and cattle. This was proven not to be the case as no effect on ruminal fermentation or the associated ruminal microbiota in either cattle or buffaloes was observed when feeding maize grains produced from crops grown on soil treated with a commercial AMF preparation. Considering the beneficial effect of AMF on maize cultivation, in terms of environmental and economic sustainability of forage production, this is clearly a positive outcome. Prokaryotic community composition, protozoal concentrations, and several ruminal fermentation parameters significantly differed between buffalo and cattle, even though they were fed and managed under the same conditions. The different nutrional requirements of these non-lactating animals may have been a contributing factor to this, along with previously reported differences in digestive physiology and rumen retention time between these two bovine species.

## Data Availability Statement

The raw sequence data generated in this study is deposited in ENA under study accession number PRJEB37928, with individual sample accession numbers ranging from ERS4525212-ERS4525234. Supplementary Material includes supplementary methods (diet composition, feed chemical analysis, rumen fermentation analysis, and DNA extraction), host nutritional requirements (Supplementary Tables 1, 2), phylum level prokaryotic community composition data for individual animals (Supplementary Figure 1), genus level prokaryotic community composition data for individual animals (Supplementary Figure 2), and weighted and unweighted UniFrac NMDS plots (Supplementary Figure 3).

## Ethics Statement

The animal study research protocol was approved by the National Ethics Committee (Ministry of Health Decree 26/2014, authorization n°399/2018, Italy) in accordance with the guidelines established by the EU Council/Directives 86/609/EEC.

## Author Contributions

AC designed and performed the experiment. DM and SD performed feed analyses and processing. GC performed the statistical analysis and interpretation of univariate data. AC together with JE performed the rumen prokaryotic community composition and microbial concentration analysis. GH performed the statistical analysis of the rumen prokaryotic community composition data. AC, JE, and GH wrote the manuscript with input from all authors. All authors approved the final manuscript.

## Conflict of Interest

The authors declare that the research was conducted in the absence of any commercial or financial relationships that could be construed as a potential conflict of interest.
