# Supplementary material for: Increasing the Sustainability of Maize Grain Production by Using Arbuscular Mycorrhizal Fungi Does Not Affect the Rumen of Dairy Cattle (Bos taurus) and Buffalo (Bubalus bubalis)
Source: Front Vet Sci. 2020 Oct 15;7:556764. doi: 10.3389/fvets.2020.556764 (PMC7593576; doi:10.3389/fvets.2020.556764)
Supplement: Supplementary file 1 [file Data_Sheet_1.docx]

Supplementary Material

# Supplementary Methods

## Diets

The TMR components (% on as fed basis) were as follows: sorghum silage (50.0%), second cut alfalfa hay (12.0%), crushed maize grain (M or C, 10.0%), barley grain (8.0%), soybean meal (8.0%), triticale grain (4.0%), wheat straw (2.4%), polyphite meadow hay (2.0%), fat (0.7%), and vitamin, mineral supplement and buffer (3.2%).

The basal diet components (% on as fed basis) were as follows: sorghum silage (63.7%), barley grain (9.80%), polyphite meadow hay (8.8%), wheat straw (5.9%), triticale grain (4.9%), crushed maize grain (3.9%), soybean meal (2.5%), vitamin and mineral supplement (1%), and fat (0.5%).

## Chemical Analysis of Feed

It was performed as follows. Dry matter (DM), crude protein (CP), crude fibre (CF), ether extract (EE) and ash were analyzed according to the methods of AOAC (AOAC, 1995). Neutral detergent fiber (NDF), acid detergent fiber (ADF) and acid detergent lignin (ADL) were analyzed according to the method described by Goering and Van Soest (1970). Non-structural carbohydrates (NSC) were calculated according to Van Soest et al. (1991). Cellulose was calculated as the difference between the ADF and ADL fractions and hemicellulose calculated as the difference between NDF and ADF fractions. Starch was quantified according to the AOAC 996.11 and AACC 76-13.01 methods (AOAC, 1995; AACC, 1976).

## Fermentation Analysis

Metabolic fermentation products (i.e., VFAs and lactate) were analyzed using high-performance liquid chromatography (HPLC) with an UV-vis detector (λ = 220 nm) and a refractive index detector (Waters, Milford, USA). The analytical column was an Aminex 85 HPX-87H (300 × 7.8 mm and 9 μm particle size) (Bio-Rad, California, USA) with a 4 × 30 mm security guard cartridge Carbo-H (Phenomenex, California, USA). Operating conditions were 40°C, under isocratic conditions, using a solution of 0.008N H_2_SO_4_ as the mobile phase (flow rate, 0.6 mL/min filtered through a 0.45 μm Millipore Teflon membrane and degassed). The liquid samples were diluted 1:1 (v/v) in H_2_SO_4_ (0.1N) and filtered through a 0.45 μm Teflon membrane before injection of 20 μL into the HPLC. External standard analytical curves were prepared at three different concentrations for each acid (i.e., lactate, acetate, propionate, isobutyrate, butyrate, isovalerate, and valerate) using pure standards (Sigma, USA) and the data fitted using weighted least-square regression.

**1.4 DNA Extraction**

DNA was extracted from the rumen homogenate using a protocol involving a combination of bead beating, Stool Transport and Recovery (STAR) buffer (Roche Diagnostics Nederland BV, Almere, The Netherlands) and the Maxwell ® 16 Instrument (Promega, Leiden, The Netherlands) as previously described with minor modifications (Van Lingen et al., 2017). Briefly, cells were pelleted by centrifugation at 15,000 *g* for 5 min at 4^o^C from 1 mL of rumen homogenate. The cell pellet was then resuspended in 700 μL of STAR buffer and transferred to a sterile screw-capped 2 mL tube (BIOplastics BV, Landgraaf, The Netherlands) containing 0.5 g of zirconium beads (0.1 mm; BioSpec Products, Inc., Oklahoma, USA). The sample was then treated in a bead beater (Mini bead-beater, BioSpec Products, Inc., Oklahoma, USA) at a speed of 5,000 rpm for 3 min, followed by incubation at 95 ^o^C with agitation (15 min and 300 rpm). The lysis tube was then centrifuged (13,000 g for 5 min at 4°C), and the supernatant transferred to a 2 mL microcentrifuge tube. STAR buffer (300 μL) was added to the remaining contents of the lysis tube, and all the previous steps starting with bead-beating repeated again. An aliquot (250 μL) of the combined supernatants from the sample lysis was then transferred into the custom Maxwell® 16 Tissue LEV Total RNA Purification Kit cartridge. The remainder of the extraction protocol was then carried out in the Maxwell® 16 Instrument according to the manufacturer’s instructions. The concentration and purity of the resulting DNA was assessed using a NanoDrop ND-1000 spectrophotometer (NanoDrop® Technologies, Wilmington, DE, USA).

# Supplementary TABLES

**Supplementary Table 1**. Indicative characteristics of rations for dry to lactating buffaloes. Milk normalized for fat (8.30 %) and protein (4.73%). (Bartocci et al., 2002)

|  | Dry | Production of normalized milk (kg/day) | | | | | |
| --- | --- | --- | --- | --- | --- | --- | --- |
|  |  | 7 | 8 | 9 | 10 | 11 | 12 |
| Advised intake  (kg DM/day) | 10.6 | 16.00 | 16.25 | 16.50 | 16.75 | 17.00 | 17.00 |
| Net energy  (milk FU/kg DM) | 0.63 | 0.74 | 0.76 | 0.79 | 0.82 | 0.85 | 0.89 |
| Crude Protein (g/kg DM) | 79 | 102 | 112 | 121 | 131 | 140 | 151 |
| NDF (g/kg DM) | 491 | 467 | 448 | 429 | 410 | 393 | 381 |
| NSC (g/kg DM) | 331 | 363 | 367 | 371 | 374 | 377 | 386 |

**Supplementary Table 2.** Indicative characteristics of rations for dry to lactating dairy cows.

|  | Dry | Production of milk (kg/day) | | | | | |
| --- | --- | --- | --- | --- | --- | --- | --- |
|  |  | 15 | 20 | 25 | 30 | 35 | 40 |
| Advised intake (Kg DM/day) | 12.0** | 17 | 18 | 19 | 20 | 21 | 22 |
| Net energy (milk FU/kg DM) | 0.65 | 0.81 | 0.85 | 0.89 | 0.93 | 0.96 | 0.98 |
| Crude Protein (g/kg DM) | 120 | 144 | 151 | 157 | 165 | 173 | 180 |
| NDF (g/kg DM) | 600 | 410 | 390 | 370 | 355 | 345 | 330 |
| NSC (g/kg DM)* | - | 202 | 224 | 235 | 246 | 258 | 270 |
| * starch+sugars, (Crovetto (2002), modified) | | | | | | | |
| ** (Mordenti (2002), modified) | | | | | | | |

# Supplementary Figures


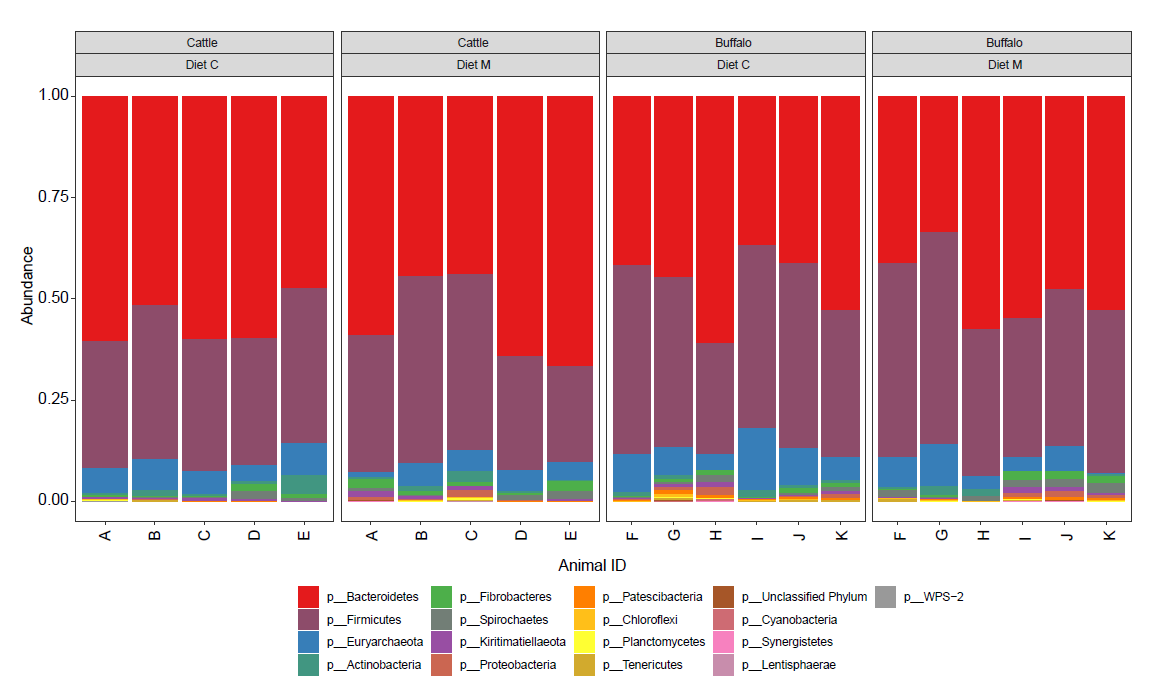


**Supplementary Figure 1:** Bar graph of the phylum level groupings of ASVs from the rumen microbiota of Holstein-Friesian cattle (animals A-E) and Mediterranean buffalo (animals F-K) fed diets that differed in terms of containing maize grain which was grown on soil that was treated (Diet M) or not (Diet C) with a commercial AMF preparation.


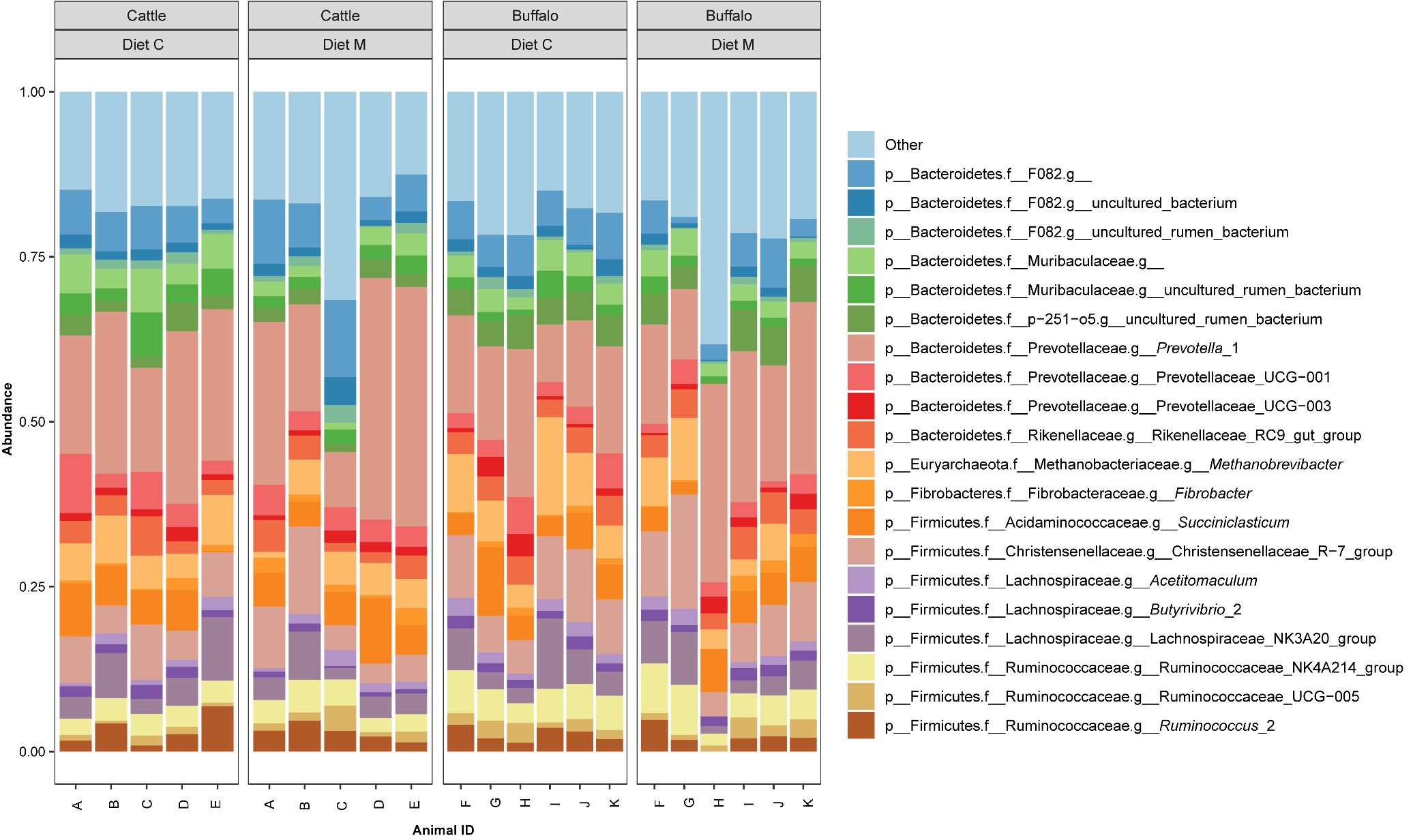


**Supplementary Figure 2:** Bar graph of the 20 most abundant genus level groupings of ASVs from the rumen microbiota of individual Holstein-Friesian cattle (animals A-E) and Mediterranean buffalo (animals F-K) fed diets that differed in terms of containing maize grain which was grown on soil that was treated (Diet M) or not (Diet C) with a commercial AMF preparation.


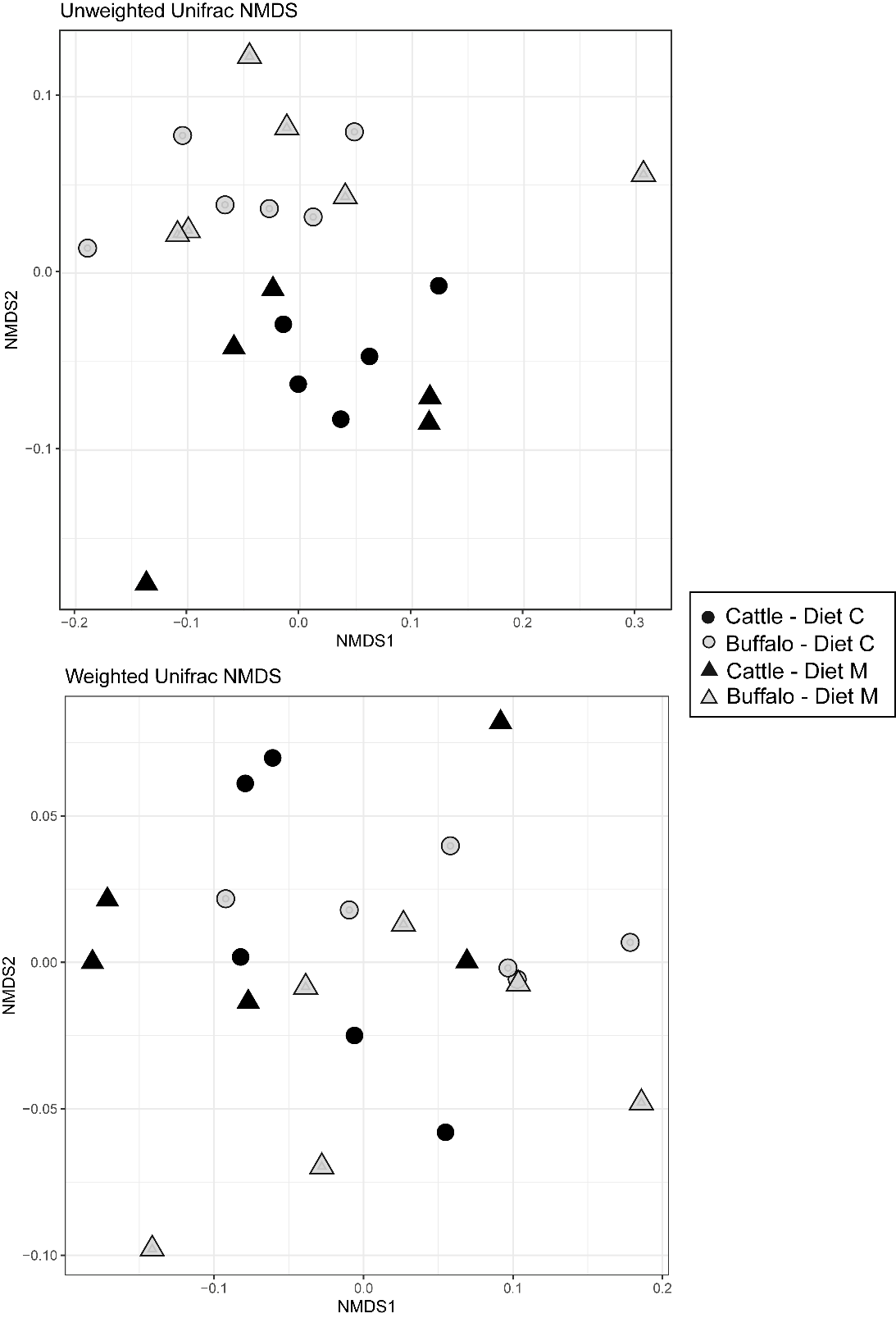


**Supplementary Figure 3:** Unweighted and weighted UniFrac analysis of betadiversity of the rumen prokaryotic community composition using unsupervised Non-metric Multidimensional Scaling.

**4 References**

AACC (1976) American Association of Cereal Chemists: “Approved Methods of the AACC”. Method 76-11

AOAC international (1995). *Official methods of analysis of AOAC international*. 16^th^ edit. Washington DC.

Bartocci S, Tripaldi C, Terramoccia S. Characteristics of foodstuffs and diets, and the quanti-qualitative milk parameters of Mediterranean buffaloes bred in Italy using the intensive system: An estimate of the nutritional requirements of buffalo herds lactating or dry. *Livestock Prod. Sci.*, (2002). 77(1), 45-58.

Crovetto GM. in: Zootecnica basi Tecnico Scientifiche. Dialma Balasini (Ed Calderini Edagricole). (2000) ISBN-10: 8820645351

Mordenti A. Regolamento per la produzione del latte del Consorzio di tutela del Parmigiano Reggiano, in: Zootecnica basi Tecnico Scientifiche Dialma Balasini (Ed Calderini Edagricole). **(**2000). ISBN-10: 8820645351

Goering HK A, van Soest, PJ. Forage fiber analyses. *U.S. Dep. Agric.*, (1970). 387–598.

van Lingen HJ, Edwards JE, Vaidya JD, van Gastelen S, Saccenti E., van den Bogert B, et al. Diurnal dynamics of gaseous and dissolved metabolites and microbiota composition in the bovine rumen. *Front. Microbiol.* (2017). **8**, 425. doi:10.3389/fmicb.2017.00425.

van Soest PJ, Robertson JB, Lewis BA. Methods for Dietary Fiber, Neutral Detergent Fiber, and Nonstarch Polysaccharides in Relation to Animal Nutrition. *J. Dairy Sci.* (1991). **74**, 3583–3597. doi:10.3168/jds.S0022-0302(91)78551-2.
